# Microwave Deposition of Palladium Catalysts on Graphite Spheres and Reduced Graphene Oxide Sheets for Electrochemical Glucose Sensing

**DOI:** 10.3390/s17102163

**Published:** 2017-09-21

**Authors:** Jian-De Xie, Siyong Gu, Houan Zhang

**Affiliations:** Fujian Provincial Key Laboratory of Functional Materials and Applications, School of Materials Science and Engineering, Xiamen University of Technology, Xiamen 361024, China; gu-siyong@163.com (S.G.); hazhang@xmut.edu.cn (H.Z.)

**Keywords:** palladium catalysts, non-enzymatic catalysis, glucose sensors, graphene oxide sheets, microwave synthesis

## Abstract

This work outlines a synthetic strategy inducing the microwave-assisted synthesis of palladium (Pd) nanocrystals on a graphite sphere (GS) and reduced graphene oxide (rGO) supports, forming the Pd catalysts for non-enzymatic glucose oxidation reaction (GOR). The pulse microwave approach takes a short period (i.e., 10 min) to fast synthesize Pd nanocrystals onto a carbon support at 150 °C. The selection of carbon support plays a crucial role in affecting Pd particle size and dispersion uniformity. The robust design of Pd-rGO catalyst electrode displays an enhanced electrocatalytic activity and sensitivity toward GOR. The enhanced performance is mainly attributed to the synergetic effect that combines small crystalline size and two-dimensional conductive support, imparting high accessibility to non-enzymatic GOR. The rGO sheets serve as a conductive scaffold, capable of fast conducting electron. The linear plot of current response versus glucose concentration exhibits good correlations within the range of 1–12 mM. The sensitivity of the Pd-rGO catalyst is significantly enhanced by 3.7 times, as compared to the Pd-GS catalyst. Accordingly, the Pd-rGO catalyst electrode can be considered as a potential candidate for non-enzymatic glucose biosensor.

## 1. Introduction

The development of sensitive and stable glucose biosensors, based on an electrochemical technique, has motivated scientists and researchers due to the practical need for monitoring blood sugar [1,2]. The aspect of electrochemistry on the progress of glucose sensors could be extended to a variety of applications including environmental protection, pharmaceutical industry, water treatment, and food science [3,4,5]. The electrochemical sensing approach has received considerable attention due to its simplicity, low detection limit, and compatibility for miniaturization [6]. Moreover, the detection limits of glucose sensors via the electrochemical route well suit the blood glucose range of healthy individuals and diabetic patients (1.1–20.8 mM) [6,7]. Based on the glucose oxidation reaction (GOR) mechanism, the glucose detection is generally divided into two types: enzymatic glucose and non-enzymatic glucose sensors [8]. To date, traditional glucose sensors on the market are mainly based on glucose oxidase-assisted electrochemical oxidation, that is, enzymatic glucose [9]. Despite its high selectivity and sensitivity, the enzymatic glucose sensor still suffers from some drawbacks such as expensive enzymes, the requirement of a complex and tedious enzyme immobilization process, and instability due to the inherent fragility of enzymes [9,10]. Accordingly, the non-enzymatic glucose sensor, based on direct electrocatalytic oxidation, has gradually been emerging and is expected to replace the enzymatic glucose sensor. As a result, it is essential to search for versatile sensing materials with ultrahigh electrocatalytic activity in GOR processes.

It is generally recognized that carbon nanomaterials are commonly used electrode materials and supports in biosensor applications. The selection of carbon support is vital for electrocatalytic reactions since the carbon support offers a synergistic effect, imparting low ionic diffusion resistance, high mechanical strength, good chemical stability, excellent charge transport capability, and good compatibility with biomaterials [11,12,13]. Moreover, both catalytic performance and catalyst utilization strongly depend on the catalyst loading and distribution uniformity on carbon supports [8]. Based on the above deductions, this work aims at the investigation on the effect of carbon support on the catalytic activity of metallic catalysts for non-enzymatic glucose sensor. We employ two kinds of carbon supports, a graphite-like sphere (GS) and a reduced graphene oxide (rGO) sheet, which are decorated with nanosized catalysts for the GOR. The GS (i.e., XC-72) is one of the most popular carbon supports for electrode applications, especially for membrane electrode assembly in fuel cells. The rGO sheets are capable of providing two-dimensional conductive layers not only for the well dispersion of catalysts, but also fast charge transfer. However, there are few reports on the performance comparison between GS and rGO sheets for the GOR activity [11].

Recently, efforts have been devoted to examining metal or metal oxide materials for non-enzymatic catalysis toward glucose detection, including Fe_2_O_3_ nanowires [10], Pd-Rh alloys [11], binary (Ni-Co)(OH)_2_ [14], cable-like CuO/Cu nanowires [15], and so on. However, the direct GOR on noble metal (e.g., Pt [16], Au [17] and Ag [18]) electrodes still earn substantial attention because of their attractive properties involving improved electrocatalytic activity and rapid response. Among noble metals, palladium is extensively employed in catalytic applications due to its advantageous properties, for example, high catalytic efficiency and excellent stability [2,19,20]. Additionally, it has been reported that the amount of Pd is more abundant, approximately 50 times more than that of Pt on the earth [21]. Accordingly, the present work adopts an efficient microwave-assisted route to synthesize Pd nanoparticles onto different carbon supports. The pulse microwave method enables the formation of crystalline Pd nuclei at low temperatures within a short period, forming high-performance catalysts toward the GOR. The catalytic GOR on the as-prepared Pd catalysts is systematically investigated by cyclic voltammetry (CV) and chronoamperometry measurement. A comparison of catalytic activity and sensitivity on the Pd catalyst electrodes is made to explore the commercial feasibility of non-enzymatic GOR in practical applications

## 2. Experimental Section

### 2.1. Pulse Microwave Synthesis of Pd Catalysts

The GS sample (Vulcan XC-72R, Cabot Co., Boston, MA, USA), commercial carbon powder, was supplied by the Cabot Corporation. Prior to the Pd deposition, the GS samples were chemically oxidized by impregnating them in 3 N nitric acid at 95 °C. The chemical oxidation enabled the implantation of oxygen functionalities onto the surface of GS, including carboxyl (O−C=O), carbonyl (C=O), and ether (C−O) groups. The rGO sheets used here were prepared by using a modified Hummers’ method, followed by a thermal reduction at 400 °C under H_2_-containing atmosphere. The GS support had a spherical-form with point-to-point contact for charge transfer, whereas the rGO sheets could provide two-dimensional conductive planes, lowering smaller charge-transfer resistance as compared to GS support. Metallic Pd catalysts were uniformly deposited on the carbon supports (i.e., oxidized GS and rGO) using microwave heating of ethylene glycol solutions of PdCl_2_ precursor salts. The carbon powders were carefully put into Pd-containing solution, which consisted of 1 mL of 0.04 M PdCl_2_, 1 mL of 0.04 M KOH, and 28 mL of diethylene glycol, in a beaker. Prior to microwave synthesis, an ultrasonic dispersion treatment was adopted to ensure good uniformity of carbon slurries. The beaker was placed into an ultrasonic bath for 1 h. Afterward, the beaker was placed into a household microwave oven (Tatung Co., Taipei, Taiwan, 900 W, 2.45 GHz), which was equipped with one *K*-type thermocouple and one temperature controller. The pulse microwave-assisted process was carried out under microwave irradiation with a power of 720 W. For safety reasons, we installed the gas safety valve in the back door of the microwave oven. One proportional-integral-derivative (PID) controller with six-step temperature program was equipped to control the real temperature of microwave oven and avoid the overheating situation. The modified microwave oven could also be operated under intermittent heating mode. Both the power-on and power-off periods were set at 3 s, and the total cycle number was 100 cycles. Thus, the microwave synthesis process for each catalyst sample took a period of 10 min. The synthesis temperature was kept at 150 °C during the growth process, controlled by the PID temperature controller. The Pd-coated carbon composites were then filtered and dried in a vacuum oven at 100 °C overnight. The Pd catalyst samples were designated as Pd-GS and Pd-rGO, based on different types of carbon supports.

### 2.2. Characterization and GOR Activity of Pd Catalysts

The crystalline structure of as-prepared Pd catalysts was analyzed by X-ray diffraction (XRD) with Cu-*K*α radiation, using an automated X-ray diffractometer (Shimadzu LabX, XRD-6000, Kyoto, Japan). A thermo-gravimetric analyzer (TGA, Perkin Elmer TA7, Waltham, MA, USA) was employed to characterize the Pd weight loading on the carbon supports. The TGA analysis was conducted in air with a heating rate of 10 °C·min^−1^ from 30 to 800 °C. The uniformity and morphology of Pd catalysts was analyzed by using high-resolution transmission electron microscope (HR-TEM, JEOL JEM-2100, Tokyo, Japan) and field-emission scanning electron microscope (FE-SEM, JEOL JSM-5600).

A drop coating method was employed to deposit as-prepared Pd powders onto carbon paper (SGL 10AA, Sigracet, Meitingen, Germany) with an area of 0.5 cm^2^. The catalyst powders were well mixed with isopropyl alcohol (12.5 mL) and 5.0 wt % Nafion (0.28 mL). The catalyst ink was carefully dropped over carbon paper to obtain catalyst electrodes. Finally, all catalyst electrodes were dried at 105 °C in a vacuum oven overnight. For comparison, commercial Pt-GS carbon paper (E-TEK, 40 wt % Pt loading) was also employed as a catalytic electrode for non-enzymatic GOR.

The CV measurement was carried out with a three-electrode configuration system under steady nitrogen flow at ambient temperature. We employed one Pt wire and Ag/AgCl electrode as counter and reference electrodes, respectively. The electrolyte used here consisted of 0.1 M NaOH + appropriate amount of glucose. The GOR analysis on as-prepared Pd catalyst electrodes was performed at different scan rates, that is, 1 and 5 mV·s^−1^. The amperometric measurement was used to figure out the GOR behavior, recorded on an electrochemical analyzer (CH Instrument, Inc., CHI 608, Bee Cave, TX, USA).

## 3. Results and Discussion

### 3.1. Physiochemical Properties of Pd Catalysts

**Figure 1:**
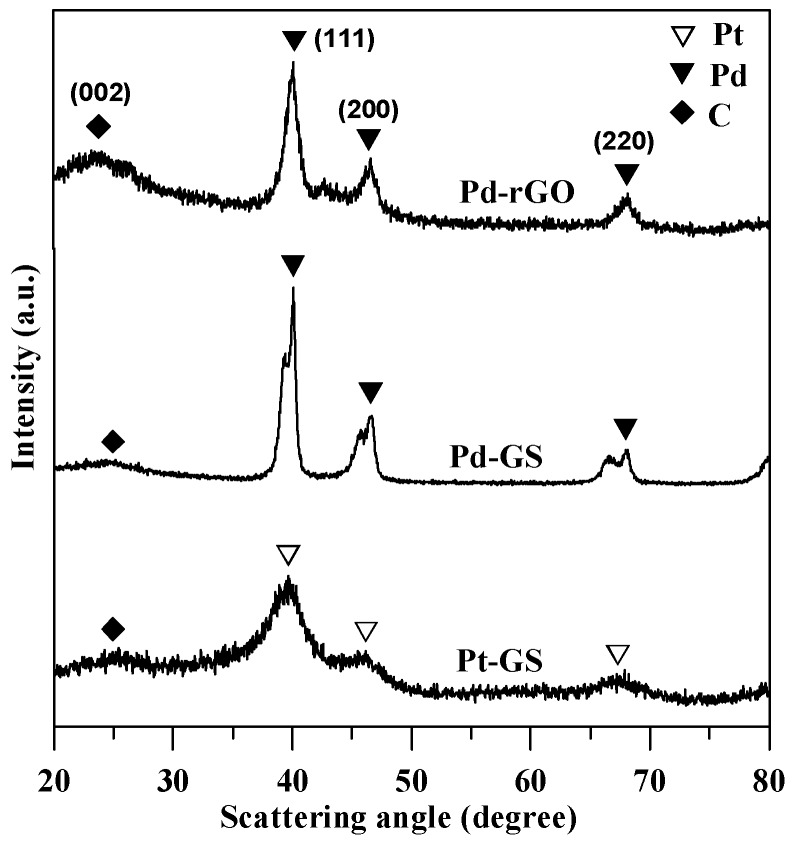
Typical X-ray diffraction (XRD) patterns of different catalyst electrodes.

Figure 1 shows typical XRD patterns of as-grown Pd and commercial Pt catalysts, revealing the major diffractions of Pd (111) and Pt (111) crystal plane of face-centered cubic (fcc) crystals. As to the Pt-GS sample, the fcc Pt-lattice diffraction peaks appear at 39.5°, 45.9°, and 67.1° for (111), (200), and (220) planes, respectively [22,23]. The representative peaks at 39.8°, 46.2°, and 67.5° can be assigned to the (111), (200), and (220) planes, respectively, of the fcc Pd lattice onto both Pd-GS and Pd-rGO samples. The XRD patterns also shows weak diffraction peaks at ca. 2*θ* = 23.5–25.6°, resulting from the graphitic structure of GS and rGO sheets. Based on the Bragg’s equation, the carbon supports display different interlayer distances (*d*_002_), that is, Pt-GS (0.348 nm), Pd-GS (0.348 nm), and Pd-rGO (0.378 nm). This result demonstrates that the pulse microwave synthesis approach is capable of synthesizing highly-crystalline Pd lattices onto different carbon supports at low temperature within a short period.

**Figure 2:**
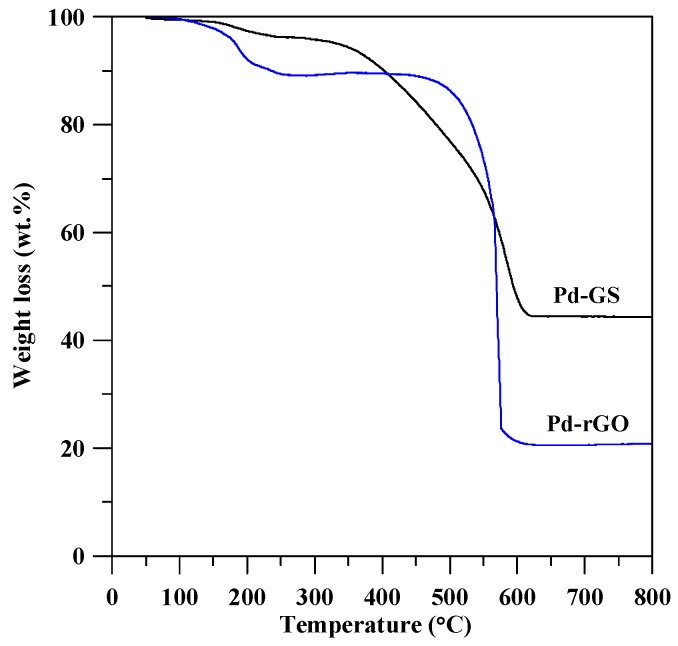
Thermo-gravimetric analyzer (TGA) weight loss curves of different catalyst electrodes.

To further evaluate the thermal stability and the amount of Pd weight loading on carbon, thermal decomposition behaviors were investigated through the TGA analysis. The TGA curves, as shown in Figure 2, clearly depict a significant mass loss for both Pd catalysts, and the maximal weight loss takes place at ca. 540 °C (Pd-GS) and ca. 570 °C (Pd-rGO). The weight loss can be attributed to the decomposition of carbon content from the carbon support, thus producing CO_2_, CO, and H_2_O gases. It can be observed from the TGA curves that there are no obvious mass losses above 600 °C. Herein the residual weight can be used to evaluate the mass percentage of metallic Pd in the as-prepared catalysts. The mass percentages of Pd crystals are calculated to be 42.3 and 20.1 wt % for Pd-GS and Pd-rGO sample, respectively. The Pt loading in Pt-GS catalyst is very close to that in Pd-GS, approximately 42.5 wt %. The weight percentages for all catalysts also deliver crucial information for examining metallic surface loading: 0.47 mg·cm^−2^ (Pt-GS); 0.45 mg·cm^−2^ (Pd-GS), and; 0.23 mg·cm^−2^ (Pd-rGO).

**Figure 3:**
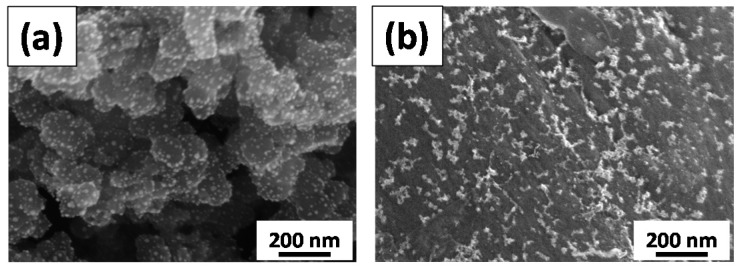
Field-emission scanning electron microscope (FE-SEM) images of different catalyst samples: (**a**) Pd-graphite sphere (GS) and (**b**) Pd-reduced graphene oxide (rGO).

**Figure 4:**
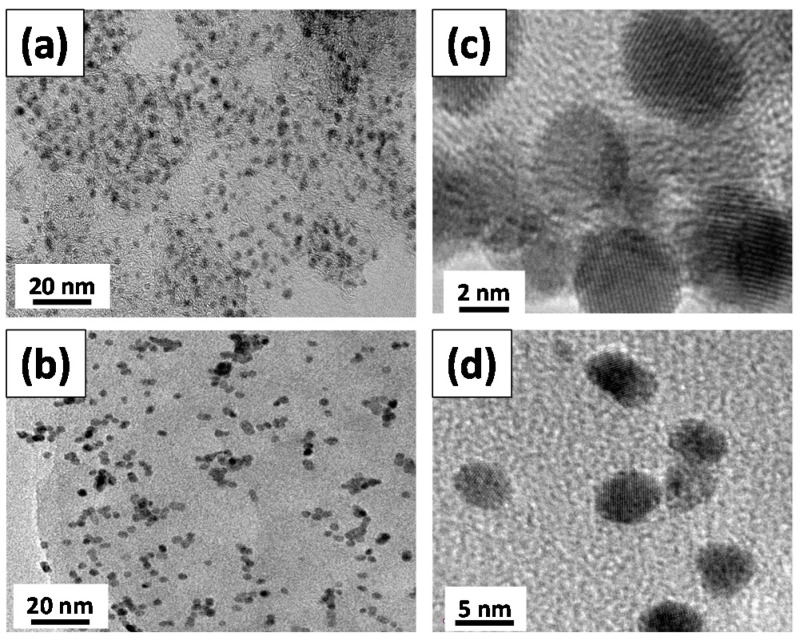
High-resolution transmission electron microscope (HR-TEM) micrographs of (**a**,**c**) Pd-GS and (**b**,**d**) Pd-rGO samples with low and high magnifications.

Figure 3 shows FE-SEM images of different catalyst samples including Pd-GS and Pd-rGO samples. Each GS has a spherical shape with an average diameter of 60 nm, whereas rGO sheets look like a flexible blanket. The resultant rGO sheets are approximately several micrometers in size, which is identical with the particle size of natural graphite powders. After the microwave synthesis of Pd deposits, a large number of bright dots represent as-grown Pd nanoparticles over the surface of carbon supports. The Pd nanoparticles are uniformly coated over both the GS and rGO sheets, forming Pd composite catalysts. Typical TEM micrographs of Pd-GS and Pd-rGO samples are illustrated in Figure 4. The as-grown Pd nanoparticles, having a spherical shape, are homogeneously dispersed over GS support without serious aggregation, while there is a slight inter-particle aggregation on the rGO sheet sample. Basically, the characteristic diameter of as-prepared Pd nanoparticles shows a narrow particle size distribution for both samples. The mean particle sizes of Pd-GS and Pd-rGO samples are approximately 5.1 and 4.8 nm, respectively. It is worth noting that the surface density of Pd onto GS support is much higher than that onto rGO sheets, identical to the result of TGA measurement. This implies that the nucleation rate of Pd crystals on the surface of GS support is superior to that on rGO sheets under microwave irradiation. As a result, the microwave synthesis imparts a rapid nucleation and crystal growth of Pd deposits under the microwave irradiation (i.e., total period of microwave synthesis: 10 min), delivering a potential to commercialization.

### 3.2. GOR Activity on Pd Catalysts

**Figure 5:**
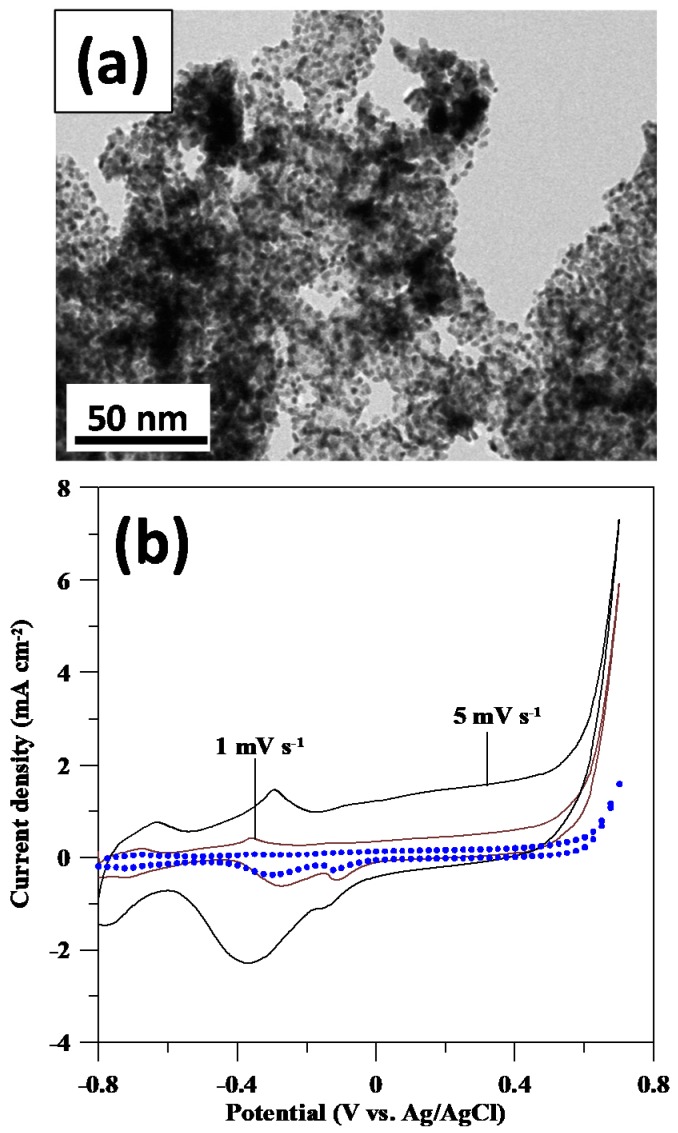
(**a**) HR-TEM micrographs of Pt-GS sample and (**b**) cyclic voltammetry (CV) profiles of Pt-GS catalyst electrode in 0.1 M NaOH + 0.1 M glucose at 1 and 5 mV·s^−1^. The symbols represent the CV profile measured at 1 mV·s^−1^ in 0.1 M NaOH without glucose.

**Figure 6:**
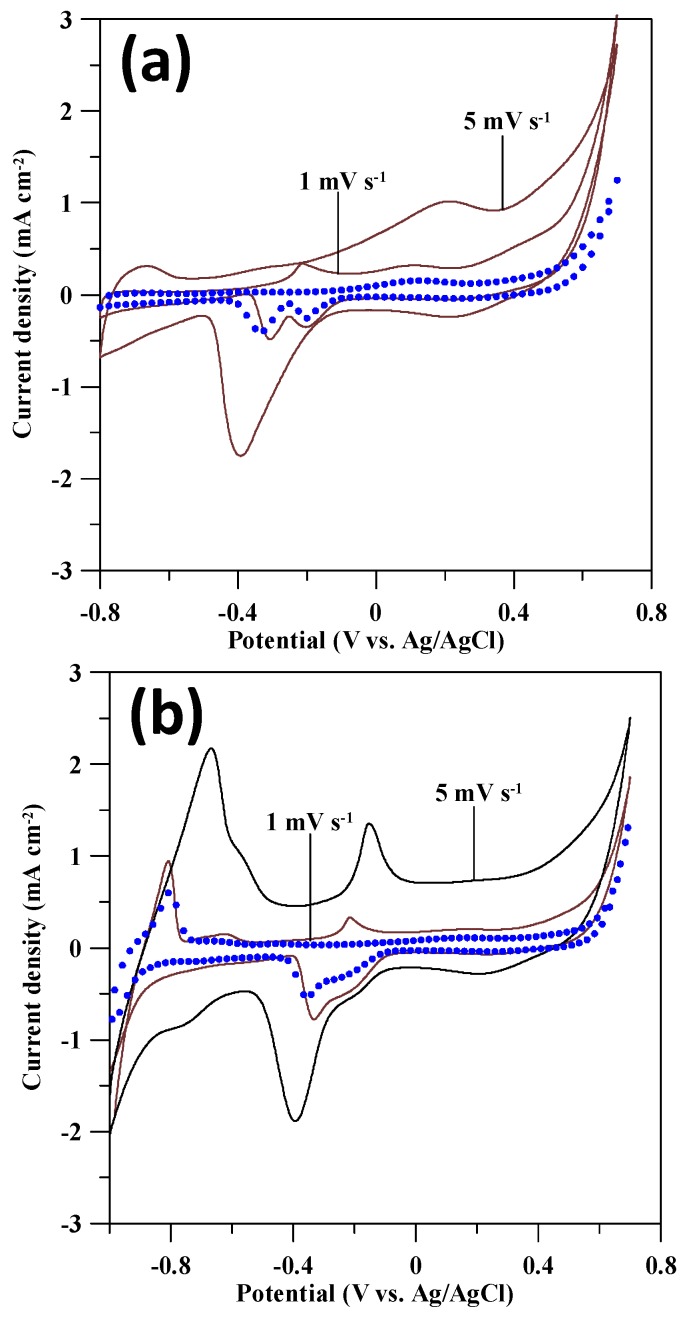
CV profiles of (**a**) Pd-GS and (**b**) Pd-rGO catalyst electrodes in 0.1 M NaOH + 0.1 M glucose. The symbols represent the CV profiles measured at 1 mV·s^−1^ in 0.1 M NaOH without glucose.

For comparison, the commercial Pt-GS catalyst was also employed for the evaluation of GOR activity. An HR-TEM photograph of Pt-GS catalyst is depicted in Figure 5a, confirming good dispersion of Pt nanoparticles onto carbon support. A large amount of Pt deposits are attached to the GS surface, and the average particle size of Pt is approximately in range from 3 to 5 nm. Due to its homogenous dispersion, there are almost no aggregations among the Pt particles. The GOR behavior on Pt-GS catalyst electrodes was investigated by using CV measurement in 0.1 M NaOH + 0.1 M glucose at 1 and 5 mV·s^−1^, as illustrated in Figure 5b. The topography of CV profiles indicates an electrochemical redox behavior in the potential range of −0.6–0 V vs. Ag/AgCl. The redox peak shows a shift to more positive potential at high scanning rate, revealing that the GOR process is controlled by the rate of heterogeneous electron transfer process. As for the GOR activity on Pd catalysts, the CV profiles of both Pd catalyst electrodes at different rates are shown in Figure 6.

The CV curves of the Pd catalyst electrodes reveal a typical feature of GOR with two oxidation peaks but different current densities. Initially, one obvious oxidation takes place, indicating that glucose molecules tend to be adsorbed on the Pd surface, thus forming an intermediate and releasing one proton from glucose. The reaction step is expressed as follows [20]:

Pd + glucose → Pd − H + intermediates(R1)

The intermediates are capable of occupying the active sites on Pd catalysts, imparting high surface coverage adsorbed by the intermediates. With the increase of potential, the Pd surface starts to form oxygenate species (e.g., −OH) in the alkali electrolyte, which is depicted as:

Pd + *x* OH^−^ → Pd–OH*_x_* + *x* e^−^ (R2)

Meanwhile, the Pd–OH*_x_* active sites serve as catalytic centers in refreshing the occupied sites (i.e., poisoned intermediates) via the following reaction steps [24]:

Pd–OH*_x_* + intermediates → Pd + glucolactone or gluconic acid (R3)

Pd–OH*_x_* + glucose → Pd + glucolactone or gluconic acid (R4)

The above GOR steps enable the regeneration of occupied sites on the Pd surface. During the negative potential scan, the reduction of Pd oxides still occurs at the potential range from −0.1 to −0.4 V vs. Ag/AgCl. Thus, the occupied sites are completely stripped and then regenerated as active sites for the sequent GOR steps [19].

**Figure 7:**
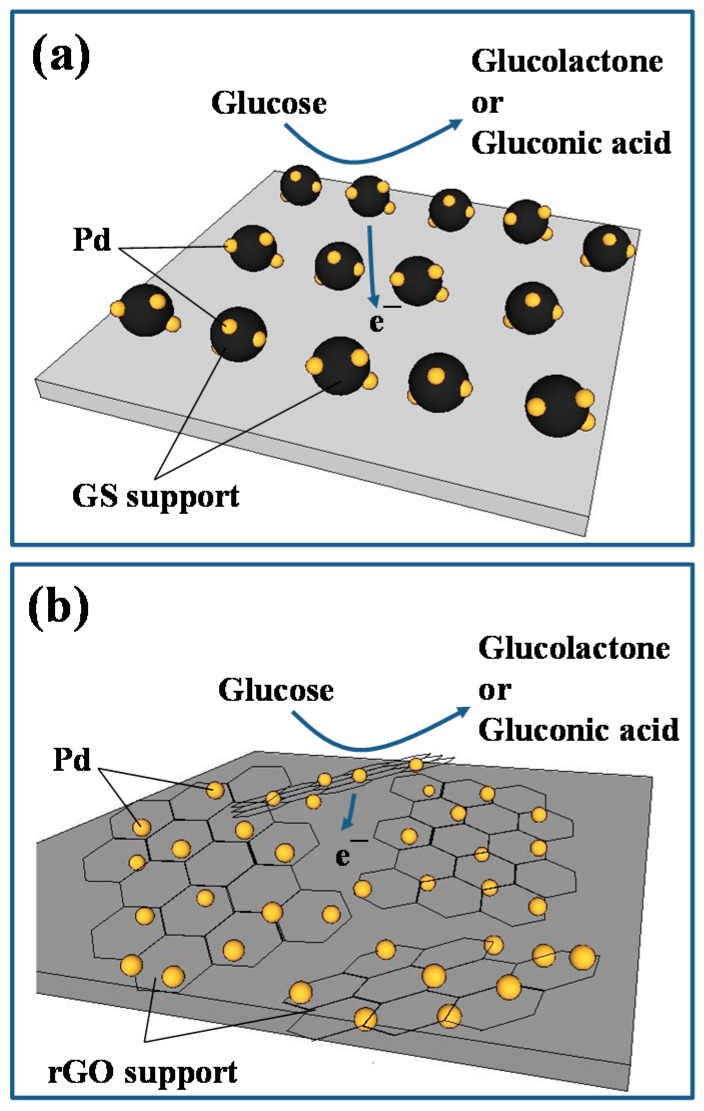
Schematic diagram for describing the GOR on (**a**) Pd-GS and (**b**) Pd-rGO catalysts.

As described by the multiple reaction steps (i.e., R1–R4), the non-enzymatic detection of glucose is strongly depended on the design of Pd catalyst electrodes. As observed from the CV profiles, the oxidation peak current is considered as an index in evaluating the specific activity of GOR on the Pd catalysts. Based on the units of metal weight, the specific current at 5 mV·s^−1^ on Pd-rGO catalyst can reach as high as 6.09 Ag^−1^, approximately three times higher than that of Pd-GS catalyst. This improved catalytic activity is attributed to the fact that the Pd-rGO catalyst electrode possesses a conductive two-dimensional network, composed of homogeneously dispersed Pd nanocatalysts. The schematic diagram for describing the GOR on both Pd-GS and Pd-rGO catalysts is briefly described in Figure 7.

The rGO support provides a two-dimensional conductive scaffold, capable of rapidly conducting electron and well dispersing Pd nanocrystals. Accordingly, the presence of rGO support not only ensures fast charge-transfer but also facilitates diffusion of glucose in liquid phase. Due to smaller Pd nanoparticles and better uniformity, Pd-rGO catalyst offers larger number of active sites for GOR. In contrast, Pd-GS catalyst electrode suffers from sluggish chemical kinetics in catalyzing glucose due to its large particle size and interrupted charge transfer (i.e., strong interfacial resistance among GS powders). As a result, the unique design of Pd-rGO catalyst allows fast ionic diffusion and rapid electron conduction, as compared to Pd-GS one.

### 3.3. Sensitivity of Pd Catalysts

**Figure 8:**
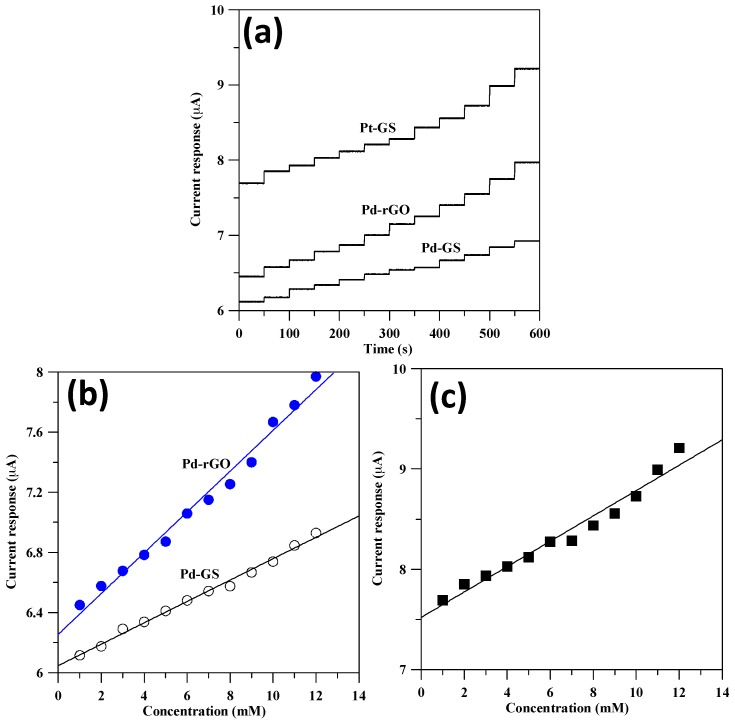
(**a**) Current response as a function of injection period of glucose under N2 atmosphere. Linear calibration curves of current response versus glucose concentration for (**b**) Pd-GS and Pd-rGO and (**c**) Pt-GS catalysts electrodes.

Figure 8a shows typical *I-t* response plots of commercial Pt and as-prepared Pd catalyst electrodes at 0.1 V and 0.3 V vs. Ag/AgCl, respectively, in 0.1 M NaOH solution with a stepwise change of glucose concentration. It can be observed that the amperometric responses basically display a step-like function in accordance with a stepwise addition of glucose. The *I-t* response plots evidently reflect fast response of glucose detection on as-prepared Pd catalyst. After 12-time stepwise dropping, the signal intensity is found to have the sequence: Pt-GS (1.52 μA) ≈ Pd-rGO (1.52 μA) > Pd-GS (0.81 μA). The signal intensity for each catalyst electrode was calculated by the gained density after the 12-time stepwise dropping. It is worth noting that the Pd weight loading in Pd-rGO catalyst is much lower than that of commercial Pt-GS and homemade Pd-GS ones. The finding reveals that the design of Pd-rGO catalyst electrode delivers high electrocatalytic activity and excellent sensitivity toward GOR. This essentially originates from a highly appropriate architecture, small Pd nanoparticles and conductive rGO sheets, facilitating electroactive surface and diffusion resistance of glucose molecules in the catalyst electrode. On the basis of experimental results, the synergistic effect incorporated with Pd nanocrystals and conductive rGO sheets plays a vital role in determining the performance of glucose detector.

The calibration curves of the amount of injected glucose and current response could be correlated and collected in Figure 8b,c. The linear plots show good correlation with the experimental data for all catalyst electrodes toward the glucose detection with the entire concentration range. The calibration curves display good linear fits within the concentration range of 1–12 mM with correlation coefficients Pt-GS (0.965), Pd-GS (0.993), and Pd-rGO (0.988). It is well known that diabetes mellitus is diagnosed by detecting a blood glucose concentration higher than normal range of 4.4–6.6 mM [9]. Thus, the glucose sensor developed by our group can be applied to determine the level of blood glucose without any further dilution treatment. The as-prepared Pd-rGO sensor presents a wider linear range as compared to other single noble metal catalyst electrodes. A comparison of non-enzymatic sensors with enzymatic glucose sensors was based on noble metal nanoparticles supported on various carbon supports, including Nafion/glucose oxidase/Pd/carbon nanofibers: 0.06–6 mM [2], glucose oxidase/Pd/chitosan/graphene: 0.001–1 mM [25], glucose oxidase/Pd@Pt nanocubes: 1–6 mM [26], Au/graphene oxide/carbon sheet: 0.05–4.9 mM [27], and Nafion/Au/Ni-Al layered double hydroxide/carbon nanotubes/graphene: 0.01–6.1 mM [28]. It can be concluded that the design of noble metal-coated catalyst electrodes achieve high sensitivity but narrow detection range. Moreover, the microwave-assisted synthesis method takes advantages involving facile preparation procedure, time saving, and without costly glucose oxidase. The sensitivity of the catalyst electrodes was analyzed by plotting the current response over the analyte concentration. The sensitivity for all catalysts is ordered as Pd-rGO (1.18 mA g^−1^·mM^−1^) > Pt-GS (0.54 mA g^−1^·mM^−1^) > Pd-GS (0.32 mA g^−1^·mM^−1^) according to the linear plots. The sensitivity was obtained based on the units of metal weight. This result reveals that the sensitivity of Pd-rGO catalyst electrode can be significantly enhanced by 3.7 times, as compared to Pd-GS catalyst. Accordingly, the Pd-rGO catalyst offers high sensitivity and superior electrocatalytic activity, mainly contributed by the synergetic effect that combines small crystalline size and two-dimensional conductive support, thereby leading to high accessibility to non-enzymatic catalysis of glucose.

## 4. Conclusions

This work outlined a synthetic strategy allowing the preparation of Pd nanocrystals on GS and rGO supports, forming the Pd catalysts toward non-enzymatic GOR. The pulse microwave approach only took a short period (i.e., 10 min) to fast synthesize Pd nanocrystals onto a carbon support at low temperature. The selection of carbon support was chosen as a crucial factor in affecting Pd particle size and dispersion uniformity. The robust design of Pd-rGO catalyst electrode delivered an enhanced electrocatalytic activity and sensitivity toward GOR, benefiting the performance of glucose biosensors. The enhanced performance was mainly attributed to the synergetic effect that combines small crystalline size and two-dimensional conductive support, imparting high accessibility to the non-enzymatic catalysis of glucose. The rGO sheets could serve as a two-dimensional conductive scaffold, capable of rapidly conducting electrons and supporting Pd nanocrystals well. The linear plot of current response versus glucose concentration was well correlated within the range of 1–12 mM. The sensitivity of Pd-rGO catalyst was significantly enhanced by 3.7 times, as compared to Pd-GS catalyst. Since the microwave synthesis method was efficient and time-saving, the as-synthesized Pd-rGO catalyst electrode could be considered as a feasible candidate for non-enzymatic glucose biosensor. Further work to study possible interferences and verify the application to real samples is in progress.
